# Chronic Fluoxetine Treatment Suppresses Plasticity (Long-Term Potentiation) in the Mature Rodent Primary Auditory Cortex *In Vivo*

**DOI:** 10.1155/2014/571285

**Published:** 2014-02-25

**Authors:** Hans C. Dringenberg, Leora R. Branfield Day, Deanna H. Choi

**Affiliations:** ^1^Department of Psychology, Queen's University, Kingston, ON, Canada K7L 3N6; ^2^Center for Neuroscience Studies, Queen's University, Kingston, ON, Canada K7L 3N6

## Abstract

Several recent studies have provided evidence that chronic treatment with the selective serotonin reuptake inhibitor (SSRI) fluoxetine can facilitate synaptic plasticity (e.g., ocular dominance shifts) in the adult central nervous system. Here, we assessed whether fluoxetine enhances long-term potentiation (LTP) in the thalamocortical auditory system of mature rats, a developmentally regulated form of plasticity that shows a characteristic decline during postnatal life. Adult rats were chronically treated with fluoxetine (administered in the drinking water, 0.2 mg/mL, four weeks of treatment). Electrophysiological assessments were conducted using an anesthetized (urethane) *in vivo* preparation, with LTP of field potentials in the primary auditory cortex (A1) induced by theta-burst stimulation of the medial geniculate nucleus. We find that, compared to water-treated control animals, fluoxetine-treated rats did not express higher levels of LTP and, in fact, exhibited reduced levels of potentiation at presumed intracortical A1 synapses. Bioactivity of fluoxetine was confirmed by a reduction of weight gain and fluid intake during the four-week treatment period. We conclude that chronic fluoxetine treatment fails to enhance LTP in the mature rodent thalamocortical auditory system, results that bring into question the notion that SSRIs act as general facilitators of synaptic plasticity in the mammalian forebrain.

## 1. Introduction

There currently is considerable interest in developing therapeutic strategies to enhance plasticity of the adult central nervous system (CNS). Physical exercise, diet, and various forms of environmental/cognitive enrichment have all been proposed to facilitate plasticity [1–4]. In addition, pharmacological approaches may offer an effective means to promote CNS plasticity. For example, the maturation and strength of GABAergic signaling act as a critical regulator of plasticity in cortical networks, with increasing inhibitory tone during postnatal development generally limiting plasticity [5–9]. Consequently, manipulations aimed at reducing the strength of GABAergic transmission are thought to constitute promising candidate strategies to enhance plasticity of mature, less plastic cortical circuits [7, 8].

Interestingly, recent reports have provided evidence for the notion that chronic treatment with the antidepressant and selective serotonin reuptake inhibitor (SSRI) fluoxetine can reduce the strength of GABAergic inhibition and promote plasticity of forebrain synapses. For example, chronic (4 weeks) fluoxetine administration restored ocular dominance shifts in the primary visual cortex (V1) of adult rats, a form of developmentally regulated plasticity that is significantly reduced in the mature brain [10]. In addition, fluoxetine treatment allowed V1 synapses to express greater long-term potentiation (LTP) [10], an electrophysiological index of the ability of synapses to undergo an upregulation of synaptic strength [11]. These plasticity-promoting effects of chronic fluoxetine administration appeared to be mediated by a decrease in intracortical inhibition and translated into significant behavioral effects, as assessed by the restoration of visual functions in a rat model of adult amblyopia [10]. Thus, chronic SSRI treatment may offer significant, therapeutic potential for the restoration of plasticity to levels normally present only during the earlier stages of postnatal brain development [7].

The notion that chronic SSRI treatment can exert a prominent, facilitating effect on plasticity has also been supported by investigations employing structural and neuroanatomical measures. Guirado et al. [12] noted that, in rats, 14-day treatment with fluoxetine resulted in an increase in immediate early gene (c-fos) expression in the somatosensory cortex, together with an increased spine density of cortical pyramidal cells; similar results have also been obtained in hippocampal pyramidal cells [13]. Finally, it is now well established that fluoxetine administration enhances neurogenesis in the hippocampal formation of adult animals [14–16], an effect that appears to be a critical mediator of some of the behavioral effects seen with SSRI treatment [15].

The evidence summarized above indicates the potential of fluoxetine to affect plasticity of forebrain synapses. It is important to note, however, that some investigations have failed to detect beneficial effects (or noted adverse outcomes) of chronic fluoxetine treatment on plasticity or in animal models of several neurological diseases, some of which clearly involve deficient plasticity mechanisms (Down syndrome, fetal alcohol syndrome, and neurotoxic brain damage) [17–20]. Consequently, there is a need for further, detailed investigations of the effects of SSRI treatment on plasticity mechanisms across various forebrain networks.

In the present study, we assessed the effect of chronic fluoxetine treatment on LTP in the thalamocortical auditory system between the medial geniculate nucleus (MGN) and primary auditory cortex (A1) of adult rats. LTP in this projection system shows a sharp, age-dependent decline over postnatal life, with high levels of LTP present during the first 5-6 weeks of postnatal life, but only modest levels after postnatal day (PD) 100 [21]. Here, we tested whether chronic fluoxetine treatment of adult rats would restore LTP to levels normally seen only in juveniles, similar to the effects reported for plasticity in V1 of adult rodents [10].

## 2. Materials and Methods

### 2.1. Animals

Experiments were conducted on adult, male Long-Evans rats (obtained from Charles River Laboratories Inc., Saint-Constant, Québec, Canada; 200–250 g body weight or about 50–55 days old at the arrival in the animal colony; at least 90–95 days old at the time of the electrophysiological procedures). Rats were individually housed (cage dimensions 40 × 20 × 20 cm) with *ad libitum* access to food and water. Individual housing was required in order to measure fluid intake for each animal. The colony room was maintained under a reversed 12 /12 hour dark/light cycle (lights on at 7 pm). Experimental procedures were performed in accordance with the published guidelines of the Canadian Council on Animal Care and approved by the Queen's University Animal Care Committee. All efforts were made in order to minimize animal suffering and the number of animals employed for these experiments.

### 2.2. Fluoxetine Treatment

All animals were allowed at least 1 week of acclimatization to the animal colony prior to the onset of fluoxetine treatment. Rats were randomly assigned to either the fluoxetine or water condition. The fluoxetine treatment regimen was the same as that described by Vetencourt et al. [10]. Fluoxetine (capsules containing 10 mg fluoxetine hydrochloride, obtained from the Kingston General Hospital Pharmacy, Kingston, ON, Canada) was dissolved in the drinking water at 0.2 mg/mL and was available ad libitum; control animals received drinking water without drug. Drinking bottles were covered with cardboard or tinfoil to prevent photodecomposition of fluoxetine and were refilled every 48 hours. Treatment continued for 4-5 weeks (*M*_period_ = 4.5 and 4.7 weeks for fluoxetine and water rats, resp.), with fluid intake (every 48 hours) and body weight (every 7 days) recorded throughout the treatment period.

### 2.3. Surgical Preparation

Electrophysiological assessments were carried out at the end of the treatment period (rat age of about 90–95 days) and followed previously established procedures [21, 22]. Rats were anesthetized using urethane (Sigma-Aldrich, Oakville, ON, Canada; 1.5 g/kg, given as three intraperitoneal (i.p.) injections of 0.5 g/kg each, every 20 min, further supplements as required to reach deep, surgical anesthesia). In addition, the local anesthetic Marcaine (0.2-0.3 mL) was administered subcutaneously under the skin covering the skull. Throughout the experiment, body temperature was maintained between 36-37°C with an electrical heating blanket.

After anesthesia induction, rats were placed in a stereotaxic apparatus, the skull was exposed, and small holes were drilled over the MGN (AP −5.5, L +4.0, V −5.4 to −6.4) and the ipsilateral A1 (AP −4.5, L +7.0, V −3.2 to 5.4, all measurements from bregma). Additional holes over the cerebellum and frontal cortex were used to secure reference and ground connections (jewelry screws), respectively. A stimulation electrode (concentric bipolar electrode, SNE-100, Rhodes Medical Instruments, David Kopf, Tujunga, CA) was lowered into the MGN, and a recoding electrode (125 *μ*m diameter Teflon-insulated stainless steel wire) was placed in A1. The final, ventral placement of both electrodes was optimized to yield the maximum amplitude of field postsynaptic potentials (fPSPs) in A1 in response to single pulse stimulation of the MGN.

### 2.4. Electrophysiology Recordings

The fPSPs in A1 were recorded differentially against the cerebellar reference connection. Signals were amplified (Model 1800, A-M Systems Inc., Carlsborg, WA, half-amplitude filter settings at 0.3 Hz to 1 kHz), digitized (at 10 kHz using a PowerLab/4s system, running Scope software v.4.0.2, AD Instruments, Toronto, ON, Canada), and stored for subsequent offline analysis. Stimulation (0.2 ms pulse duration) of the MGN was delivered by means of a stimulus isolation unit (ML 180 Stimulus Isolator, AD Instruments).

For each rat, a 30 min period following the final electrode adjustments was given to allow for stabilization prior to the onset of data collection. Following stabilization, an input-output curve was generated by stimulating the MGN at successively increasing intensities (0.1–1.0 mA, in 0.1 mA increments). The intensity that elicited fPSPs of 50–60% of the maximal fPSP amplitude was chosen for the formal data collection.

Baseline fPSPs were recorded every 30 s until a 30 min period of stable baseline recordings was established. Subsequently, theta-burst stimulation (TBS) of the MGN was applied as trains of 10 bursts (delivered at 5 Hz), with each burst consisting of five pulses at 100 Hz. Stimulation trains were repeated every 10 s for a total of four trains. This induction protocol has previously been shown to elicit reliable LTP in the thalamocortical auditory system *in vivo* [21, 22]. After the TBS delivery, fPSPs were recorded for 60 min (every 30 s), followed by a second TBS episode (same as above) and a final 60 min of fPSP recordings.

### 2.5. Histology

Immediately after the experiment, rats were perfused through the heart with 0.9% saline, followed by 10% formalin. Brains were extracted and immersed in formalin prior to sectioning (40 *μ*m) using a cryostat. The locations of all electrodes were examined using standard histological techniques and only animals with accurate placements were included in the analysis of the electrophysiology data.

### 2.6. Data Analysis

Data are expressed as mean ± standard error of the mean (SEM). The fPSP amplitude was computed offline by Scope software (v.4.1.1, AD Instruments). Values for each rat were averaged over 10 min intervals and these averages were normalized by dividing them by the averaged baseline (pre-TBS) amplitude of that animal. Data were statistically evaluated by repeated measures analysis of variance (ANOVA) and, if statistically appropriate, simple effects tests using the CLR ANOVA software package (v.1.1, Clear Lake Research Inc., Houston, TX). The level of significance for statistical analyses was set at *P* < 0.05. Note that the results of all statistical analyses are reported in the appropriate figure captions.

## 3. Results

### 3.1. Body Weight and Water Intake

**Figure 1:**
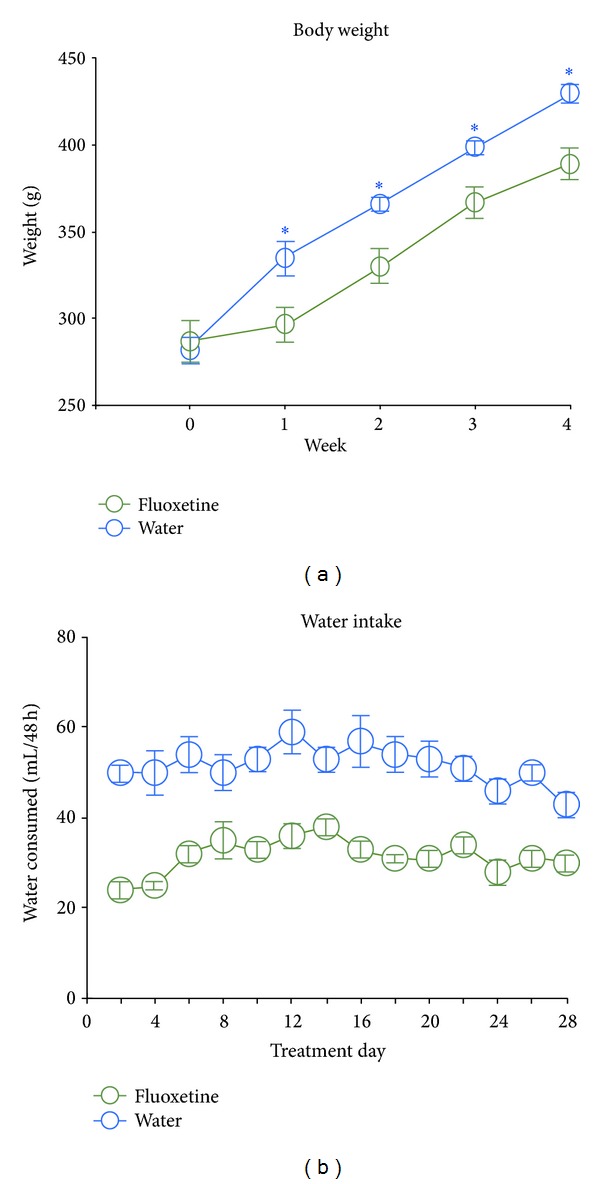
Body weight and water intake of rats given access to drinking water (*n* = 18) or drinking water containing fluoxetine (0.2 mg/mL; *n* = 20) during a 4-week treatment period. (a) Fluoxetine treatment significantly reduced body weight gain during the treatment period. ANOVA results: effect of time, *F*(4,144) = 370.8, *P* < 0.0001; effect of group, *F*(1,36) = 5.8, *P* = 0.02; interaction, *F*(4,144) = 13.8, *P* < 0.0001; * indicates significant (*P* < 0.05) simple effects tests. (b) Fluoxetine also reduced fluid intake relative to the control (water) condition during the entire treatment period. ANOVA results: effect of time, *F*(13,286) = 2.3, *P* = 0.006; effect of group, *F*(1,22) = 43.9, *P* < 0.0001; interaction, *F*(13,286) = 0.9, *P* = 0.6. Note that statistics for fluid intake are limited to *n* = 12 for both groups since some of the water bottles used did not permit accurate measurement of fluid consumption.

For a 4-week period, rats were given access to drinking water (*n* = 18) or drinking water containing fluoxetine (0.2 mg/mL; *n* = 20). During this period, weight gain was significantly reduced in rats given access to fluoxetine relative to control (water) animals (Figure 1(a)). The total weight gain from Week 0 (before treatment onset) to Week 4 was 37 ± 3% and 54 + 3% for fluoxetine and water animals, respectively, findings that are consistent with the substantial literature demonstrating appetite-suppressant effects of fluoxetine treatment [19, 20, 23]. Fluoxetine also reduced water intake (Figure 1(b)), with fluid consumption over 48 h averaging 31 ± 1 mL and 51 ± 3 mL in fluoxetine and water animals, respectively.

### 3.2. Electrophysiology

**Figure 2:**
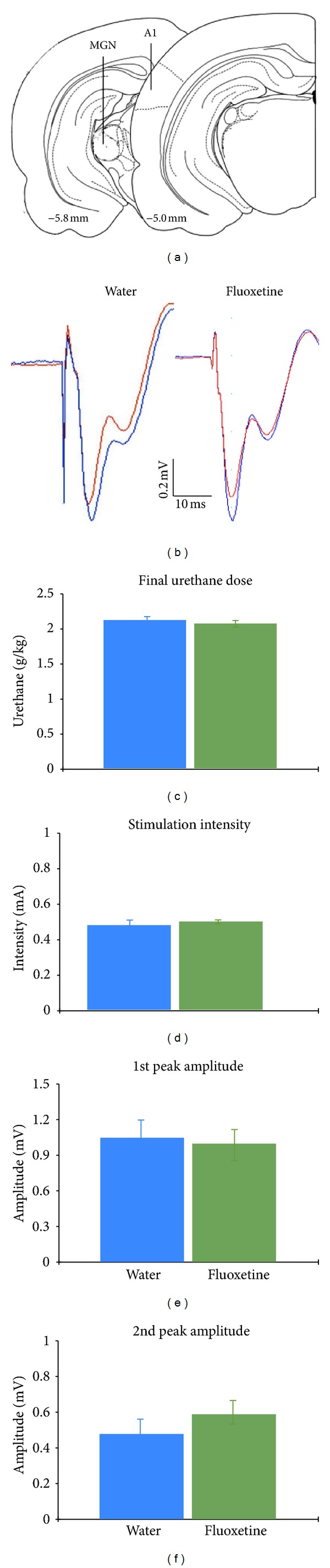
(a) Schematic diagram of electrode placements in the medial geniculate nucleus (MGN) and primary auditory cortex (A1); numbers indicate distance (in mm) from bregma. Diagrams adapted from Paxinos and Watson [40]. (b) Field postsynaptic potentials (fPSPs) recorded in A1 in response to single pulse MGN stimulation consisted of two successive, negative-going peaks. (the initial, sharp, negative spike is the stimulation artifact.) Red and blue traces are taken before and after theta-burst stimulation of the MGN, respectively. Note the absence of clear potentiation of the second fPSP peak in the fluoxetine-treated rat. (c) Final urethane dose required for anesthesia induction in the two treatment groups. (d) Stimulation intensities used for the electrophysiological experiments. (e) Baseline (pre-LTP induction) amplitude of the first fPSP peak. (f) The second fPSP peak recorded in A1 of rats given access to drinking water (*n* = 11) or water containing fluoxetine (0.2 mg/mL; *n* = 14) during a 4-week treatment period. Statistical analyses for all comparisons did not reveal any significant group differences. (*P*'*s *>0.05; note that the 23% amplitude increase for the second fPSP peak in fluoxetine animals also failed to approach statistical significance, *P* = 0.3.)

After 4-5 treatment weeks (4.5 and 4.7 weeks for fluoxetine and water animals, resp.), each rat was anesthetized with urethane to allow for the placement of a stimulating and recording electrode in the MGN and A1, respectively (Figure 2(a)). Consistent with previous wok [21, 22], extracellular recordings in the middle layers (III/IV) of A1 revealed that single pulse stimulation of the MGN elicited fPSPs consisting of two negative-going components with peak latencies of about 6–8 and 14–16 ms, respectively (Figure 2(b)). Previous work using current-source density analysis and pharmacological approaches has revealed that these two negative peaks correspond to current sinks associated with the sequential activation of direct, thalamocortical synapses (layer IV; first fPSP peak) and subsequent, intracortical synapses (layers II/III; second fPSP peak) [22, 24]. It is important to note that the urethane dose required for deep, surgical anesthesia did not differ significantly between the two groups of animals, with water and fluoxetine rats receiving a final dose of 2.12 ± 0.09 and 2.07 ± 0.06 g/kg of urethane, respectively (Figure 2(c)). Thus, chronic fluoxetine treatment did not appear to alter the response to urethane anesthesia.

Initially, fPSPs elicited by single-pulse MGN stimulation were recorded for 30 min in order to establish a measure of baseline synaptic strength prior to LTP induction. Stimulation intensities used for the two groups of animals did not differ significantly, with MGN stimulation pulses of 0.48 ± 0.04 mA and 0.5 ± 0.02 mA for water (*n* = 11) and fluoxetine (*n* = 14) animals, respectively (Figure 2(d)). Further, the amplitude of baseline fPSPs did not differ between the treatment groups, with the amplitude of the first peak at 1.05 ± 0.15 mV and 1.0 ± 0.15 mV in water and fluoxetine animals, respectively (Figure 2(e)). The amplitude of the second fPSP peak was 0.48 ± 0.07 mV and 0.59 ± 0.08 mV in the water and fluoxetine group, respectively (Figure 2(f)). It is noteworthy that the amplitude difference between the two groups amounts to 23%, even though the statistical analysis did not indicate this effect to approach significance (*P* = 0.3).

**Figure 3:**
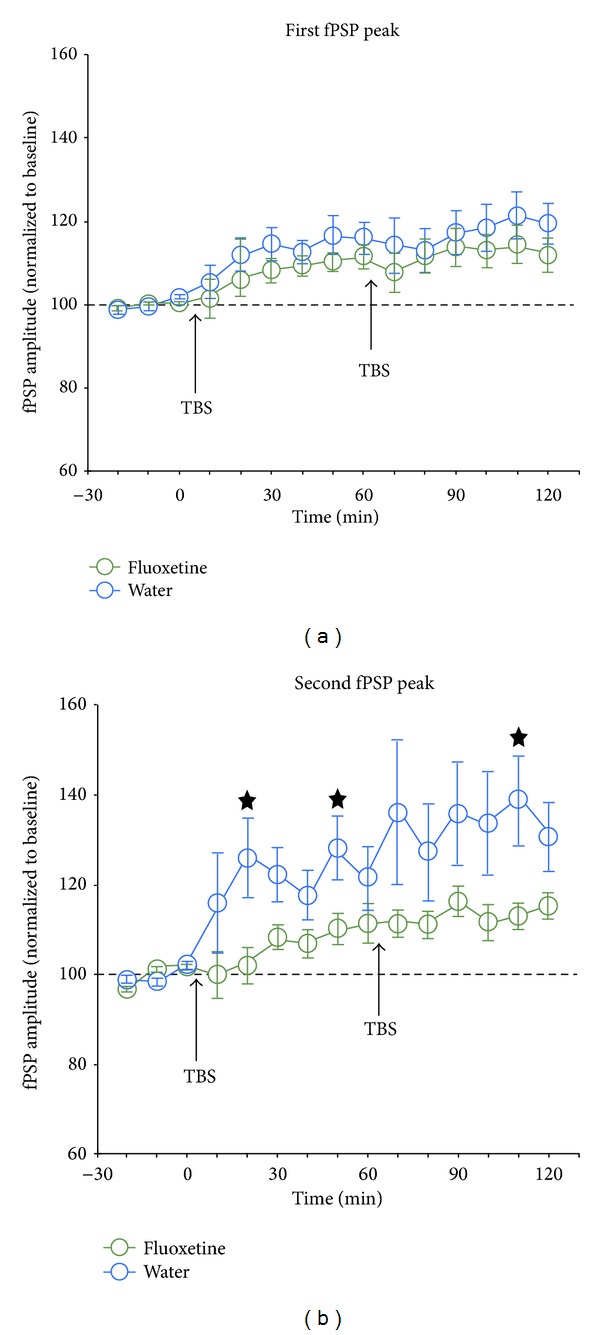
Amplitude of the first (a) and second (b) peak of field postsynaptic potentials (fPSPs) in the auditory cortex of rats treated with water (*n* = 11) or fluoxetine (*n* = 14), and the effect of theta-burst stimulation (TBS, at arrow) of the medial geniculate nucleus. Two episodes of TBS resulted in significant increases of amplitude of both the first (a) and second (b) fPSP peak in both groups of rats. However, for the second fPSP peak, fluoxetine-treated rats showed significantly less LTP than that seen in water animals. ANOVA results for (a) effect of time, *F*(14,322) = 10.4, *P* < 0.0001; effect of group, *F*(1,23) = 0.8, *P* = 0.4; interaction, *F*(14,322) = 0.5, *P* = 0.9. ANOVA results for (b) effect of time, *F*(14,252) = 10.0, *P* < 0.0001; effect of group, *F*(1,18) = 3.6, *P* = 0.08; interaction, *F*(14,252) = 2.2, *P* = 0.007; * indicates significant (*P* < 0.05) simple effects tests comparing the two treatment groups. (Note that group sizes for the analysis of the second peak were *n* = 9 and *n* = 11 for water and fluoxetine rats, resp., since this peak could not be consistently resolved in all animals.)

Following the completion of baseline recordings (30 min), two episodes of TBS were delivered to the MGN, each followed by 60 min of fPSP recordings. In water animals, TBS resulted in successful LTP induction, with the first TBS episode resulting in a potentiation of the first and second fPSP peak to 115% and 123% of baseline, respectively (Figure 3; all values reported here are mean values for recordings taken between 31–60 min after TBS delivery). The second TBS episodes resulted in further potentiation in water animals, with the two peaks reaching 120% and 135% of baseline (Figure 3).

In fluoxetine animals, TBS also resulted in LTP, but the amplitude of the first and second fPSP peak reached only 111% and 110% of baseline, respectively, following the first TBS episode (Figure 3). Further, after the second TBS, both peaks reached only 113% of baseline (Figure 3). Thus, fluoxetine animals showed less potentiation than that seen in the water group, an effect that was significant for the second fPSP peak representing intracortical synapses (see caption for Figure 3).

**Figure 4:**
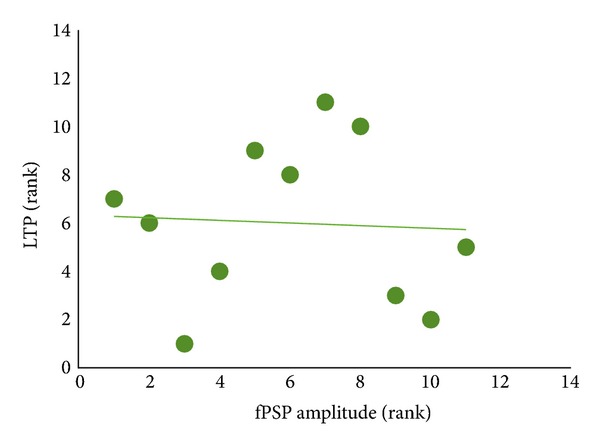
Baseline (pre-theta-burst stimulation) amplitude of the second field postsynaptic potentials (fPSPs) peak and levels of long-term potentiation (LTP) in rats treated with fluoxetine (*n* = 11). Data are expressed as rank, with Rank 1 indicating the highest fPSP amplitude and greatest level of LTP. Note the absence of a significant correlation (*P* > 0.05) between the two variables.

As mentioned, the second fPSP peak in fluoxetine rats exhibits a baseline amplitude 23% higher than that seen in water animals. Even though this effect did not reach statistical significance, it might nevertheless indicate a minor enhancement of intracortical synaptic strength following chronic fluoxetine exposure. Such an enhancement may act to limit (occlude) further LTP induction by TBS delivery. In order to assess this possibility, we performed additional analysis by plotting and correlating baseline fPSP amplitude against levels of LTP for the second fPSP peak in all fluoxetine animals (Figure 4). However, correlations using either raw or rank-ordered data (Figure 4) both failed to indicate a significant relation between baseline fPSP amplitude and subsequent potentiation induced by TBS in fluoxetine rats.

## 4. Discussion

With the present experiments, we examined whether chronic fluoxetine treatment alters LTP of synapses in the mature A1 of adult rats. Several recent reports have provided support for the notion that chronic fluoxetine treatment leads to an enhancement of plasticity in the adult CNS [7, 10, 12–14, 25]. In contrast to these findings, we found no evidence of an upregulation of plasticity at A1 synapses. In fact, there was a clear suppression of LTP in fluoxetine-treated rats, in particular for the second peak of the cortical fPSP, thought to reflect synaptic currents originating at intracortical synapses in A1 (see below).

Previous work has shown that, in rats, fPSPs elicited by MGN stimulation *in vivo* typically consist of two distinct, negative peaks that correspond to current sinks associated with the successive activation of thalamocortical and intracortical A1 synapses, respectively [22, 24, 26]. For both sets of synapses, LTP induced by thalamic stimulation shows a clear, age-related decline, with significant potentiation present up to PD 50, modest LTP around PD 100 (about the time LTP was assessed in the present study), and very little or no LTP after PD 200 [21, 24]. As such, LTP in the rat thalamocortical auditory system provides an appropriate model to study the developmental decline of plasticity in a central sensory system.

The present data confirm that adult rats show modest levels of plasticity under the present experimental conditions, with thalamocortical and intracortical synapses expressing LTP of about 120% and 135% of baseline, respectively. Surprisingly, rats given chronic fluoxetine showed less LTP, with both thalamocortical and intracortical synapses expressing potentiation of only 113% of baseline, respectively, observations that are indicative of an inhibition of LTP induction mechanisms, especially for intracortical synapses in A1.

The results summarized above regarding LTP in control animals are consistent with previous work, which has also shown that intracortical synapses in the adult A1 show higher LTP levels relative to thalamocortical synapses [21, 22, 24]. Interestingly, receptive field plasticity of A1 neurons (i.e., shifts in the optimal response to different sound frequencies) also occurs by a potentiation of intracortical but not thalamocortical synapses [27], suggesting that the LTP measured here plays a direct role in receptive field plasticity and associated changes of the tonotopic map present in A1. Consequently, it is possible that chronic fluoxetine exposure may also impair A1 receptive field shifts in rodents, a hypothesis that clearly requires examination.

We employed the same fluoxetine dosing regimen (0.2 mg of fluoxetine in 1 mL of drinking water) as that used in previous work showing a restoration of ocular dominance plasticity and enhancement of LTP in V1 slice preparations obtained from adult rats [10]. In the present investigation, rats in the fluoxetine condition consumed an average of about 15.5 mL of fluid every 24 hours, equaling an intake of about 3.1 mg of fluoxetine. This drug amount corresponds to a daily dosage of about 10.7 mg/kg and 7.9 mg/kg of body weight at the beginning (body weight of about 290 g) and the end (390 g) of the treatment period, respectively. These dosages are very similar to those used in previous chronic administration studies demonstrating behavioral and/or neurochemical effects following fluoxetine treatment [10, 17, 19, 28]. In our experiments, fluoxetine reduced body weight gain during the treatment period, a classic effect of SSRI treatment [17, 19, 20, 23]. In addition, we also noted a suppression of water intake, also consistent with the results of previous work [28]. Together, these results confirm the bioavailability and bioactivity of fluoxetine, as administered in the present investigation.

While fluoxetine has been suggested to enhance plasticity of the mature CNS, empirical evidence assessing this contention has been inconsistent. Stewart and Reid [17] noted that 15-day treatment with fluoxetine reduced levels of LTP in the dentate gyrus of anesthetized rats, and inhibitory effects on hippocampal (area CA1) LTP in rats have also been reported for acute (single-dose) fluoxetine treatment [29]. An elegant, recent investigation revealed that a chronic (4 week) fluoxetine regimen resulted in deficits in the induction of LTP in the hippocampal CA1 field of adult rats, while dentate gyrus LTP was intact [30]. Interestingly, the same authors also noted a disruption of LTD, which again was specific for the CA1 field [30]. The LTD impairment is of significance since it suggests that any observed reduction in LTP is unlikely to be related exclusively to an upregulation of synaptic strength following long-term fluoxetine exposure (see [30]). Typically, such an effect reduces LTP but yields greater LTD, due to the fact that enhanced synaptic connectivity represents a potentiated state, which limits further potentiation but leaves greater room for synaptic weakening [31–33]. It will be important for future work to assess whether chronic fluoxetine impairs or facilitates LTD induction in areas other than the hippocampal formation.

The present experiments did not provide reliable evidence for an upregulation of A1 synaptic strength following chronic fluoxetine, with baseline fPSP amplitudes showing no significant differences between the two treatment groups. Previous work has found evidence for an enhancement of field potential strength in rats after fluoxetine administration in both the dentate gyrus and CA1 field [17, 30], even though a lack of fPSP facilitation in the rat dentate gyrus has also been reported [18]. It is noteworthy, however, that the second fPSP peak was 23% larger in fluoxetine rats relative to water animals, in particular since this value is very similar to the reduction of LTP for the second fPSP peak in the fluoxetine group (135% and 113% potentiation in water and fluoxetine rats, resp., 22% difference). Despite the nonsignificance of the baseline fPSP difference, we carried out additional analyses to assess whether greater baseline fPSP amplitude was related to lower levels of TBS-induced LTP, suggestive of an occlusion-like effect of chronic fluoxetine in LTP. However, these analyses did not provide any suggestion of an association between baseline fPSP amplitude and subsequent LTP magnitude. We cannot rule out that such a relation, or a significant difference in baseline synaptic strength, may emerge with larger sample sizes or alternative methodologies to study synaptic connectivity and plasticity in A1 (e.g., optical imaging or *in vitro* approaches). It is also possible that some CNS regions (e.g., hippocampal formation) exhibit greater sensitivity to the potential, neurotrophic effects elicited by fluoxetine than areas such as the primary sensory fields of the neocortical mantle.

Previous work has shown that local application of an NMDA receptor antagonist directly in A1 of rats blocks the induction of LTP elicited by MGN stimulation *in vivo* [22, 34]. It is well documented that the precise subunit composition of the NMDA receptor exerts profound effects on LTP induction by altering the level of calcium influx across the postsynaptic membrane. Receptors containing the NR2B subunit exhibit prolonged channel opening duration and greater calcium influx relative to NR2A-expressing receptors, effects that lead to enhanced LTP induction [21, 22, 35–37]. Interestingly, in rats, chronic fluoxetine exposure can alter NMDA subunit composition by increasing the relative expression of NR2A subunits [38], raising the possibility that this effect contributes to the reduction of LTP noted here and in previous work [17, 30]. Future work is required to examine this hypothesis and delineate the precise mechanisms that mediate the effects (facilitating and inhibitory) of long-term fluoxetine exposure on the induction of different forms (e.g., structural and physiological) of CNS plasticity.

In recent years, there has been considerable excitement regarding the potential of various SSRIs to enhance CNS plasticity [7, 8, 10, 14, 39]. Not only have the plasticity-promoting actions been suggested to mediate mood-enhancing effects [14–16, 39], but SSRIs may also facilitate plasticity and functional recovery in neurological conditions unrelated to mood disorders (e.g., [7, 10, 18–20]). While some evidence is clearly supportive of this notion [10, 12, 13, 25], the present study confirms and extends previous investigations that have failed to detect an enhancement of plasticity following chronic fluoxetine treatment [17, 30]. In fact, both electrophysiological (LTP) and structural investigations are compatible with the view that fluoxetine exposure can result in a strengthening and stabilization of synaptic connectivity, which reduces the ability of neurons to express further plasticity [12, 13, 30, 38]. Thus, the effects of fluoxetine on plasticity appear to be complex and bidirectional, findings that clearly require consideration when discussing the use of fluoxetine and other SSRIs for the modulation of plasticity of the mammalian forebrain.

## Abbreviations

A1: Primary auditory cortex
ANOVA: Analysis of variance
CNS: Central nervous system
fPSP: Field postsynaptic potential
i.p.: Intraperitoneal
LTD: Long-term depression
LTP: Long-term potentiation
MGN: Medial geniculate nucleus
PD: Postnatal day
V1: Primary visual cortex
SSRI: Selective serotonin reuptake inhibitor
SEM.: Standard error of the mean
TBS: Theta-burst stimulation.
